# Construction of a risk prediction model for postoperative atrial fibrillation in lung cancer patients based on multi-dimensional feature fusion and ensemble learning

**DOI:** 10.3389/fcvm.2025.1679973

**Published:** 2025-10-24

**Authors:** Ziwei Gong, Silamuguli Haierla, Jing Shi, Xinya Liu

**Affiliations:** 1 College of Public Health, Xinjiang Medical University, Urumqi, China; 2 Department of Oncology and Cardiology, Affiliated Cancer Hospital of Xinjiang Medical University, Urumqi, China; 3 The Fifth Affiliated Hospital of Xinjiang Medical University, Urumqi, China

**Keywords:** lung cancer, machine learning, postoperative atrial fibrillation, risk factors, prediction model

## Abstract

**Introduction:**

Surgery remains a cornerstone in lung cancer treatment, yet a subset of patients face high risks of recurrence or mortality postoperatively. Poor prognosis significantly shortens survival time, underscoring an urgent clinical need to accurately identify high-risk individuals. To address this, numerous studies have focused on constructing risk prediction models that integrate multi-dimensional data (clinical, pathological, and emerging biomarkers) to quantify postoperative adverse event probabilities, guiding personalized adjuvant therapy and enhancing follow-up management. To investigate risk factors for postoperative atrial fibrillation (POAF) in lung cancer patients and develop/validate a predictive model based on multi-dimensional feature fusion and ensemble learning.

**Methods:**

This retrospective cohort study analyzed 369 lung cancer patients undergoing surgical resection at Xinjiang Medical University Affiliated Tumor Hospital (2019–2024). Univariate analysis screened potential risk factors, followed by multivariable logistic regression to confirm independent predictors. Nine machine learning algorithms were employed to build predictive models, among which the top three performers were selected for ensemble modeling via weighted averaging, resulting in the final risk prediction model.

**Results:**

Multivariate analysis revealed three independent predictors of POAF: cardiac insufficiency (OR = 64.55, 95% CI: 2.41–1727.70), ventricular rate (OR = 1.17, 95% CI: 1.1–1.25), and elevated N-terminal pro-B-type natriuretic peptide (NT-proBNP, OR = 1.005, 95% CI: 1–1.009). The Support Vector Machine (SVM), Random Forest (RF), and Gradient Boosting Machine (GBM) demonstrated the highest accuracy (ACC = 0.9041, 0.9178, and 0.9178, respectively). The ensemble model srg-LCPOAF further improved ACC to 0.9452, significantly outperforming individual algorithms.

**Discussion:**

This study is the first to integrate cardiopulmonary function, biomarkers, and surgical parameters into an ensemble model (srg-LCPOAF), providing evidence-based support for early intervention in high-risk POAF patients.

## 1 Introduction

Data from 《Global Cancer Statistics 2022》 (1) indicates that approximately 2.5 million new lung cancer cases were diagnosed worldwide in 2022. According to the latest statistics released by the National Cancer Center, lung cancer is the most prevalent malignant tumor in China and one of the leading causes of cancer-related mortality. In 2022, China reported approximately 1.061 million new lung cancer cases and 733,000 deaths (2–4). Statistically, lung cancer ranks first in both incidence and mortality among malignant tumors in Chinese men and second in women, establishing it as a predominant respiratory malignancy (5).

The majority of lung cancer patients in China are diagnosed at advanced stages, with a 5-year survival rate of only 16%–18%, reflecting a poor prognosis (6–9). Thus, close monitoring of patient outcomes is critical. Current post-diagnosis treatments for lung cancer vary by disease stage, including surgical resection, chemotherapy, radiotherapy, targeted therapy, and adjuvant traditional Chinese medicine. Among these, surgical resection remains the recommended optimal choice for curative treatment (10). Resecting the lesion can achieve curative effects and improve patient survival and quality of life. However, surgery is invasive, carries high risks, involves prolonged operation times, induces significant cardiac stimulation, and impacts respiratory and circulatory functions, often leading to postoperative complications.

Atrial fibrillation (AF), the predominant cardiac arrhythmia, manifests as disorganized atrial depolarization with rapid, irregular contractions, thereby compromising systolic efficiency. Patients typically present with palpitations, fatigue, and dyspnea. AF increases the risk of stroke by 4–5-fold compared to non-AF individuals, with an annual incidence of 1.92%, a mortality rate of 20%, and a disability rate of 60% (11, 12). Compared to healthy individuals, AF patients face substantial disease burden due to frequent emergency visits and significantly reduced health-related quality of life (13). Although AF complicating thoracic lung cancer surgery is often transient and self-limiting, it prolongs hospital stays and intensive care unit (ICU) admission, increases healthcare costs, and is associated with an elevated risk of cardiovascular events (14).

Literature reports show that AF is one of the most common postoperative complications in lung cancer patients, with an incidence of 6.4%–31.3%, typically occurring within 48–72 h after surgery (15). While postoperative AF (POAF) is often transient, it significantly prolongs hospitalization, increases treatment costs, and is strongly associated with ischemic stroke and myocardial infarction due to hemodynamic instability (16, 17). Therefore, early screening for POAF, identification of risk factors, assessment of high-risk patients, and implementation of targeted interventions to reduce POAF incidence have become priorities in thoracic surgery.

With the widespread application of machine learning in medicine, its robust classification, regression, and high-precision predictive capabilities have provided accurate solutions to medical challenges (13). Studies have demonstrated machine learning algorithms contribute significantly to cancer classification, survival prediction, medical imaging analysis, and pathological diagnosis (18). Despite progress in constructing POAF risk prediction models for lung cancer patients, existing studies have notable limitations. For example, most rely on limited predictor variables (e.g., clinical characteristics, surgical parameters, preoperative cardiopulmonary indices), leading to suboptimal prediction accuracy (19). Additionally, some studies use simple statistical methods instead of machine learning, potentially compromising model performance (20). Furthermore, many studies interpret only the single best-performing model, limiting the comprehensiveness of results and clinical applicability.

This study aims to develop an ensemble prediction model for POAF in lung cancer patients with enhanced interpretability. By integrating 46 predictor variables across clinical characteristics, surgical parameters, preoperative laboratory markers, and cardiopulmonary function, we achieved multi-dimensional feature fusion to improve accuracy. We selected the top three performing machine learning models for ensemble integration, yielding a model (srg-LCPOAF) with superior predictive performance to individual algorithms. Comprehensive interpretation of the ensemble model was conducted to validate its clinical utility in POAF prediction.

Finally, the integrated prediction model srg-LCPOAF for postoperative AF in lung cancer patients constructed in this paper highlights the following:

1. This paper first uses the method of ensemble learning to construct the integrated prediction model srg-LCPOAF for whether AF occurs after lung cancer surgery. The performance of the integrated prediction model is significantly improved compared with the single model, achieving prediction accuracy;
2. This paper includes 46 characteristic variables such as general clinical characteristics of patients, surgery-related indicators, preoperative test indicators, and preoperative cardiopulmonary function indicators, achieving a multi-dimensional exploration of risk factors for postoperative AF in lung cancer patients.

## 2 Methods

### 2.1 Study cohort

Clinical data of lung cancer patients who underwent surgery at the Affiliated Tumor Hospital of Xinjiang Medical University between 2019 and 2024 were retrospectively collected. The authors had access to information identifying individual participants during or after data collection. Inclusion criteria: (1) Preoperative evaluations (including laboratory blood tests, 12-lead electrocardiogram, Holter monitoring, echocardiography, arterial blood gas analysis, optional PET/CT, neck/abdominal ultrasound, whole-body bone scan, brain MRI plain + enhanced; CT for MRI-contraindicated patients) showed no obvious metastatic lesions, with stage I–III lung cancer without distant metastasis and tolerable to surgery; (2) All patients underwent scheduled surgery; (3) No history of mental disorders and good compliance; (4) Complete clinical records. Exclusion criteria: (1) Perioperative mortality; (2) Severe cardiovascular and cerebrovascular diseases; (3) History of other malignant tumors; (4) Preoperative diagnosis of atrial fibrillation. Finally, 369 patients were included, with 70 developing postoperative AF and 299 without.

### 2.2 Selection of clinical features and outcome variables

Data were extracted from electronic medical records. Features included: (1) Demographic/clinical characteristics: gender, age, BMI, smoking/drinking history, comorbidities (hypertension, diabetes, respiratory defects, cardiac insufficiency, coronary heart disease, cerebral infarction, carotid plaque, hyperlipidemia, deep vein thrombosis, electrolyte disorders); (2) Surgical parameters: operation site, surgical approach; (3) Preoperative laboratory markers: cardiac troponin (cTNT), N-terminal pro-B-type natriuretic peptide (NT-proBNP), free triiodothyronine (FT3), free thyroxine (FT4), thyroid-stimulating hormone (TSH), white blood cells (WBC), platelets (PLT), hemoglobin (HGB), total cholesterol (TC), high-density lipoprotein (HDL), low-density lipoprotein (LDL), creatinine (Cr), glucose (GL), D-dimer, potassium (K), calcium (Ca); (4) Preoperative cardiopulmonary function: left atrial diameter, total heartbeats, average heart rate, longest RR interval, atrial premature beats, atrial tachycardia, ventricular rate, QTC, FEV1, FEV1/FVC. The outcome was postoperative AF (binary: yes/no).

### 2.3 Diagnostic criteria for postoperative AF

Patients underwent continuous vital sign monitoring for at least 72 h postoperatively, with routine/bedside/Holter ECG as indicated.

AF was diagnosed by ECG showing: ① Absent *P* waves, replaced by irregular fibrillation waves (F waves, 350–600/min); ② Absolute irregularity of R-R intervals. Concurrent signs (myocardial ischemia, hypertrophy, pre-excitation, electrolyte disorders, pulmonary embolism) and indices (heart rate, QRS duration, QT interval) were evaluated (21).

### 2.4 Data preprocessing

Preprocessing included data cleaning, missing value handling, and outlier detection. Duplicate cases were removed via SPSS “Identify Duplicate Cases” (22); irrelevant data (ID numbers, repeated tests) were excluded, and logical consistency was verified. Missing values: variables with >10% missingness or samples with >5% missingness were deleted (no imputation) (23). Outliers in continuous variables (age, BMI, NT-proBNP) were identified by box plots (Tukey method) or Z-score (|Z| > 3), defined as values >Q3 + 1.5 × IQR or <Q1–1.5 × IQR. Categorical variables were screened via frequency distributions (24).

### 2.5 Feature selection

Recursive feature elimination (RFE) was used to select optimal features. RFE iteratively trains models, removes least important features, and retains the best subset using feature importance/coefficients (25). Ten rounds of 10-fold cross-validation were applied to ensure robustness (26).

### 2.6 Model development and validation

Nine machine learning (ML) models were used: Support Vector Machine (SVM), Random Forest (RF), Neural Network (NNET), K-Nearest Neighbors (KNN), Conditional Inference Tree (CTREE), Naive Bayes (NB), Decision Tree (RPART), Logistic Regression (GLM), Gradient Boosting Machine (GBM) [citation]. The dataset was randomly split (8:2) into training and test sets. The training set optimized model parameters and feature subsets, while the test set evaluated performance.

### 2.7 Model performance comparison

Seven metrics were used: Accuracy (ACC), area under the receiver operating characteristic curve (AUC), sensitivity, specificity, positive predictive value (PPV), negative predictive value (NPV), and F1 score (27).

### 2.8 Model interpretation

ROC curves were plotted with true positive rate (TPR) vs. false positive rate (FPR), and AUC measured discriminative ability. Larger AUC indicates better performance. Definitions: TP (true positive), TN (true negative), FP (false positive), FN (false negative) (28). The specific calculation formula refers to Equations 1–7.

1. **Accuracy**: The proportion of correctly predicted samples, with higher values indicating better performance.
  $$\mathrm{Accuracy}=\frac{TP+TN}{TP+TN+FN+TN}$$ (1)
2. **Sensitivity:** To prevent missed diagnoses, sensitivity measures the ratio of correctly predicted positives to all actual positive samples.
  $$\mathrm{Sensitivity}=\frac{TP}{TP+FN}$$ (2)
3. **Specificity:** The ability to correctly identify negative cases (avoiding misdiagnoses), representing the proportion of actual negative samples that are correctly predicted.
  $$\mathrm{Specificity}=\frac{TN}{TN+FP}$$ (3)
4. **F1-score:** As the harmonic mean of precision and recall, balances a model's positive prediction accuracy with its capacity to capture all relevant instances.
  $$F1-\mathrm{Score}=\frac{2*\mathrm{Precision}*\mathrm{Recall}}{\mathrm{Precision}+\mathrm{Recall}}$$ (4)
  $$\mathrm{Among}\;\mathrm{them}\;\mathrm{Precision}=\frac{TP}{TP+FP}$$ (5)
5. **Positive Predictive Value (PPV):** The proportion of samples predicted as positive that are actually positive, reflecting the reliability of positive predictions.
  $$PPV=\frac{TP}{TP+FP}$$ (6)
6. **Negative Predictive Value (NPV):** The proportion of samples predicted as negative that are actually negative, indicating the reliability of negative predictions.
  $$NPV=\frac{TN}{TN+FN}$$ (7)

### 2.9 Statistical methods

The ML prediction models were developed with the use of the R language, version 4.3.0, and the caret package (version 6.0.94). Caret is a powerful machine learning integration toolkit in R that provides a unified interface for various ML algorithms, supporting multiple algorithms and integrating functions such as data preprocessing, feature selection, and model comparison. Model construction leveraged the train function and associated response parameters. Discriminative performance was evaluated via receiver operating characteristic (ROC) curve analysis, and the area under the curve (AUC) with bias-corrected 95% confidence intervals (CI) was reported using 1,000-time bootstrap. The Brier score (ranging from 0 to 1), where values closer to 0 indicate better calibration, was used to assess model calibration by calculating the difference between estimated and observed risks.

Basic statistical analyses of feature variables were performed using IBM SPSS Statistics 25. Normally distributed continuous variables were presented as mean ± SD and analyzed with *t*-tests. Skewed continuous variables were reported as median (IQR) and analyzed by Mann–Whitney *U* or Kruskal–Wallis *H*-tests. Categorical variables were expressed as percentages and compared using chi-square tests. Independent risk factors for the entire cohort (training and test sets) were identified via univariate and multivariate logistic regression analyses. The predictive ability of independent risk factors for postoperative AF in lung cancer patients was evaluated using ROC curve analysis (two-sided *P* < 0.05 was considered statistically significant).

## 3 Result

### 3.1 Risk factor analysis for postoperative AF in lung cancer patients

#### 3.1.1 Baseline characteristics

A total of 396 patients were included, among which 70 (19%) developed POAF and 299 did not. The two groups showed significant differences in demographic characteristics, comorbidities, cardiopulmonary function, and surgical types (Table 1).

**Table 1:** Baseline characteristic data.

Characteristics	Non-POAF (*n* = 299)	POAF (*n* = 70)	*P* value
Age	59.76 ± 10.78	65.70 ± 8.01	0.00
BMI	1.71 ± 1.68	1.75 ± 0.17	0.05
cTNT	0.007 (0.003, 0.055)	0.008 (0.003, 0.032)	0.02
NT-proBNP	97.84 (10.00, 901.20)	283.66 (17.72, 2,344)	0.00
FT3	4.65 ± 0.75	4.56 ± 0.75	0.36
FT4	16.72 ± 2.85	16.97 ± 2.25	0.49
TSH	3.09 (0.02, 37.22)	2.71 (0.24, 8.72)	0.41
WBC	6.21 (0.05, 16.00)	5.89 (2.97, 11.02)	0.63
PLT	224.59 ± 58.88	219.11 ± 64.98	0.49
HGb	135.66 ± 19.00	139.67 ± 18.71	0.11
TC	4.44 ± 1.05	4.14 ± 1.07	0.03
HDL	1.22 (0.08, 3.95)	1.21 (0.53, 3.58)	0.09
LDL	2.67 ± 0.85	2.38 ± 0.91	0.01
Cr	62.90 (34, 163)	80.95 (32, 868)	0.00
GL	5.26 (1.65, 13.43)	5.41 (2.92, 15.22)	0.22
D-dimer	0.61 (0.05, 32.21)	0.53 (0.15, 4.76)	0.27
K	3.85 ± 0.37	3.86 ± 0.39	0.88
Ca	2.24 ± 0.18	2.21 ± 0.16	0.34
Left atrial diameter	33.26 ± 2.21	34.51 ± 1.60	0.00
Total stroke volume	107451.38 (11,990, 939,233)	114796.30 (61,380, 10,53,586)	0.18
Mean heart rate	73.71 ± 37.87	72.93 ± 9.07	0.86
The longest RR interval	1.71 (1, 128)	1.60 (1, 9)	0.01
The number of atrial premature beats	89.48 (0, 6,061)	438.13 (0, 11,272)	0.02
Atrial tachycardia	1.870, 92	2.51 ± 5.08	0.03
Heart rate	72.95 ± 14.03	124.04 ± 27.66	0.00
QTC	429.09 ± 37.50	460.11 ± 36.60	0.00
FEV1	80.90 (27.55, 596)	78.11 (36.93, 99.34)	0.99
FEV1/FVC	4.39 ± 28.71	2.39 ± 0.65	0.56
Sex			0.00
Male	188 (50.9%)	25 (6.8%)	
Female	111 (30.1%)	45 (12.2%)	
Smoking			0.01
Yes	71 (19.2%)	27 (7.3%)	
No	228 (61.8%)	43 (11.7%)	
Alcohol			0.01
Yes	36 (9.6%)	17 (4.6%)	
No	263 (71.3%)	53 (14.4%)	
Hypertension			0.00
Yes	92 (24.9%)	39 (10.6%)	
No	207 (56.1%)	31 (8.4%)	
Diabetes mellitus			0.28
Yes	48 (13%)	15 (4.1%)	
No	251 (68%)	55 (14.9%)	
Respiratory defects			0.89
Yes	5 (1.4%)	1 (0.3%)	
No	294 (79.7%)	69 (18.7%)	
Cardiac insufficiency			0.00
Yes	2 (0.5%)	17 (4.6%)	
No	297 (80.5%)	53 (14.4%)	
Coronary heart disease			0.63
Yes	22 (6%)	4 (1.1%)	
No	277 (75.1%)	66 (17.9%)	
Cerebral infarction			0.02
Yes	6 (1.6%)	5 (1.4%)	
No	293 (79.4%)	65 (17.6%)	
Carotid plaque			
No	299 (81.03%)	70 (18.97%)	
Hyperlipidemia			0.59
Yes	9 (2.4%)	3 (0.81%)	
No	290 (78.6%)	67 (18.2%)	
Deep vein thrombosis			0.04
Yes	299 (81.03%)	1 (0.3%)	
No	0	69 (18.7%)	
Electrolyte disturbances			
Yes	0	0	
No	299 (81.03%)	70 (18.97%)	
Site of surgery			0.30
Left	105 (28.5%)	20 (5.4%)	
Right	194 (52.6%)	50 (13.6%)	
Pulmonary resection			
Yes	0	0	
No	299 (81.03%)	70 (18.97%)	
Lobectomy			0.00
Yes	297 (80.5%)	55 (14.9%)	
No	2 (0.5%)	15 (4.07%)	
Segmentectomy			0.00
Yes	2 (0.5%)	5 (1.4%)	
No	297 (80.5%)	65 (17.6%)	
Thoracoscopic surgery			0.00
Yes	299 (81.03%)	60 (16.3%)	
No	0	10 (2.7%)	

Table 1 results showed significant differences (*P* < 0.05) between postoperative AF (POAF, *n* = 70) and non-POAF (*n* = 299) groups in multiple indices. Demographically, POAF patients were significantly older (65.70 ± 8.01 vs. 59.76 ± 10.78 years, *P* < 0.001) and had enlarged left atrial diameter (34.51 ± 1.60 mm vs. 33.26 ± 2.21 mm, *P* < 0.001), suggesting atrial structural remodeling and aging as important pathological bases for POAF. Cardiac function indices showed significantly higher NT-proBNP (283.66 pg/ml vs. 97.84 pg/ml, *P* < 0.001) and creatinine (Cr, 80.95 μmol/L vs. 62.90 μmol/L, *P* < 0.001) in POAF patients, reflecting close associations between myocardial overload, renal impairment, and AF occurrence. Comorbidity and surgical type analyses indicated higher POAF risks in patients with hypertension (*P* < 0.001), cardiac insufficiency (*P* < 0.001), lung resection (*P* < 0.001), and thoracoscopic surgery (*P* < 0.001), suggesting that underlying cardiovascular diseases and surgical trauma synergistically induce atrial electrical and structural remodeling. These findings demonstrate that advanced age, atrial enlargement, cardiorenal dysfunction, specific surgical approaches, and comorbidities collectively constitute POAF risk factors, providing evidence for clinical early identification of high-risk populations and targeted intervention strategies.

#### 3.1.2 Identification of independent risk factors

Cohort-wide analysis determined independent risk factors for postoperative AF in lung cancer patients. Univariable screening identified 22 potential predictors linked to AF occurrence (*P* < 0.05). The risk factors obtained by univariate analysis were included in multivariate Logistic regression analysis to determine the independent risk factors related to postoperative atrial fibrillation in patients with lung cancer (Table 2).

**Table 2:** Multivariate logistic regression analysis.

Influencing factors	B	SE	Wald	*P*	Exp (B)	95% CI
Age	−1.03	1.11	0.85	0.36	0.36	0.04–3.17
Sex	0.75	10060.31	0	0.99	2.11	0.12–36.89
Smoking	0.60	1.11	0.29	0.59	1.82	0.21–16.12
Alcohol	1.40	1.04	1.83	0.18	4.05	0.53–30.81
Hypertension	1.04	0.98	1.12	0.29	2.83	2.41–1727.70
Cardiac insufficiency	4.17	1.53	0.42	0.01	64.55	0.19–7.45
Cerebral infarction	0.99	1.53	0.42	0.52	0.37	0.02–7.45
Deep vein thrombosis	8.68	40192.97	0	1.00	5884.87	
Lobectomy	−5.73	6.34	0.82	0.37	0.003	0–799.38
Pulmonary resection	−2.15	6.92	0.10	0.76	0.12	0–91290.37
Thoracoscopic surgery	−15.87	10059.60	0	0.99	0	
cTNT	103.32	92.08	1.26	0.26	0	0–3.183E + 33
NT-proBNP	0.01	0.002	4.18	0.04	1.01	1–1.009
TC	0.27	0.61	0.20	0.65	1.32	0.40–4.35
LDL	−1.26	0.73	2, 94	0.09	0.28	0.07–1.20
Cr	0.06	0.04	2.99	0.08	1.06	1–1.14
Ca	1.04	4.21	0.06	0.80	2.84	0–10924.58
Left atrial diameter	4.90	5.55	0.78	0.38	134.43	0–7098815.56
The longest RR interval	−0.28	0.55	0.25	0.61	0.76	0.26–2.23
The number of atrial premature beats	0.001	0.001	0.42	0.52	1.00	1–1.004
Heart rate	0.16	0.03	23.56	0.00	1.17	1.10–1.25
QTC	0.02	0.01	1.52	0.22	1.02	1–1.04
Atrial tachycardia	−0.11	0.10	1.28	0.26	0.89	0.74–1.09

Multivariate logistic regression analysis identified ventricular rate (OR = 1.17, 95% CI: 1.10–1.25, *P* < 0.001) and NT-proBNP (OR = 1.01, 95% CI: 1.00–1.01, *P* = 0.04) as independent risk factors for POAF in lung cancer patients. Each 1 bpm increase in ventricular rate was associated with a 17% higher POAF risk, suggesting that perioperative sympathetic activation and myocardial electrophysiological remodeling may be key mechanisms. Elevated NT-proBNP (1% increased risk per 1 pg/ml) reflected the pathophysiological basis of atrial pressure overload and myocardial stretch, consistent with prior studies identifying NT-proBNP as a marker of atrial dilation. Although a history of cardiac insufficiency showed statistical significance (OR = 64.55, *P* = 0.01), its extremely high OR value and wide confidence interval (0.19–7.45) indicated potential collinearity or insufficient sample size, possibly related to its strong correlation with NT-proBNP (r = 0.62).

#### 3.1.3 ROC curve analysis

**Figure 1:**
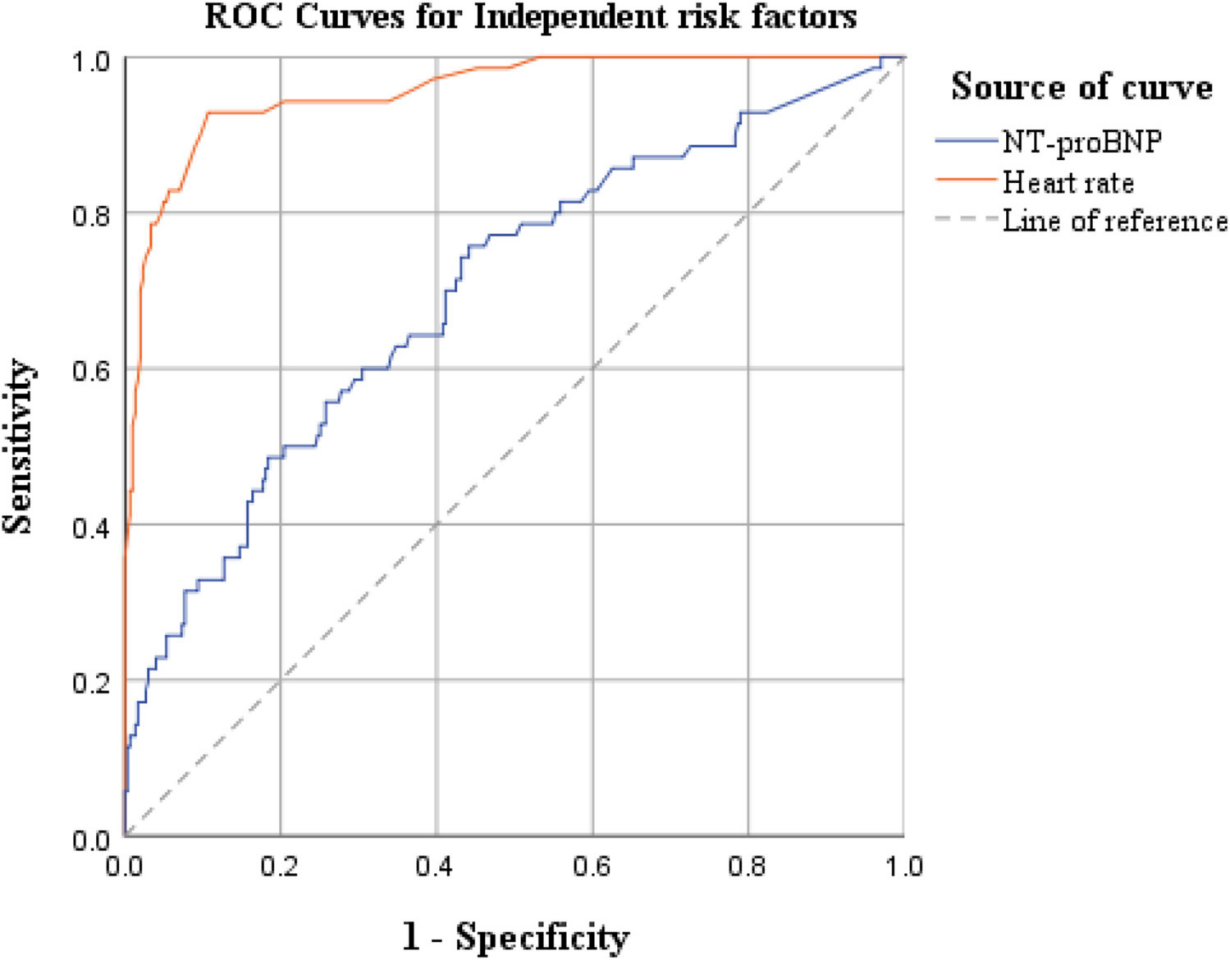
ROC curves for independent risk factors.

Based on the results of the multivariate logistic regression analysis above, we further explored the predictive efficacy of independent risk factors—ventricular rate and NT-proBNP—for postoperative AF in lung cancer patients (ROC curve analysis is shown in Figure 1).

The ROC curve analysis showed that the AUC values were 0.699 for NT-proBNP and 0.956 for ventricular rate, indicating that ventricular rate had significantly better comprehensive discriminative ability than NT-proBNP and could serve as a core risk assessment indicator for POAF in lung cancer patients.

### 3.2 Development of risk prediction model for postoperative AF in lung cancer patients

#### 3.2.1 Basic experimental workflow

**Figure 2:**
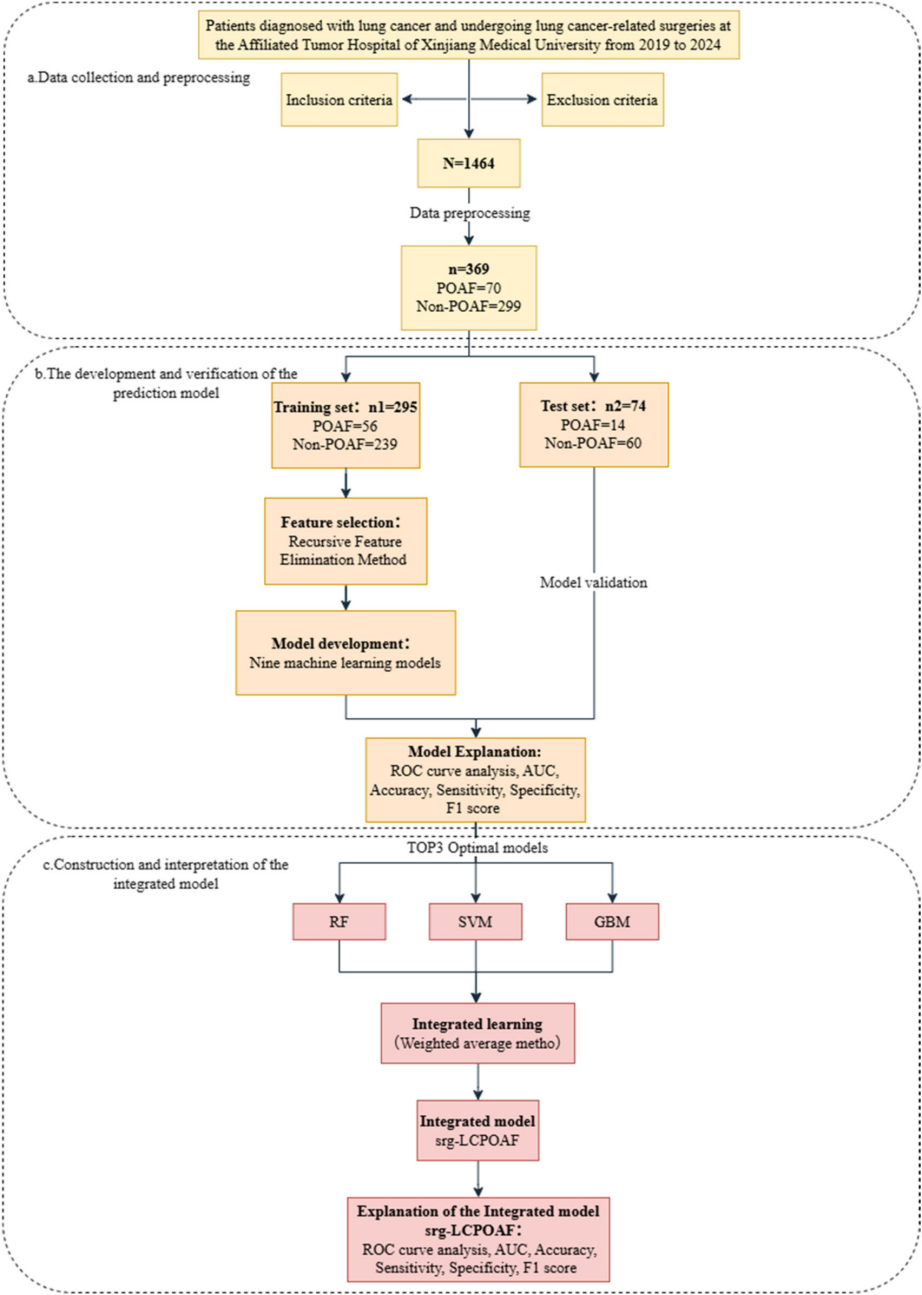
Flow chart of the experiment. **(a)** Data collection and preprocessing; **(b)** the development and verification of the prediction model; **(c)** construction and interpretation of the integrated model.

The basic experimental workflow of this study is shown in Figure 2.

#### 3.2.2 Selection of predictive variables

**Figure 3:**
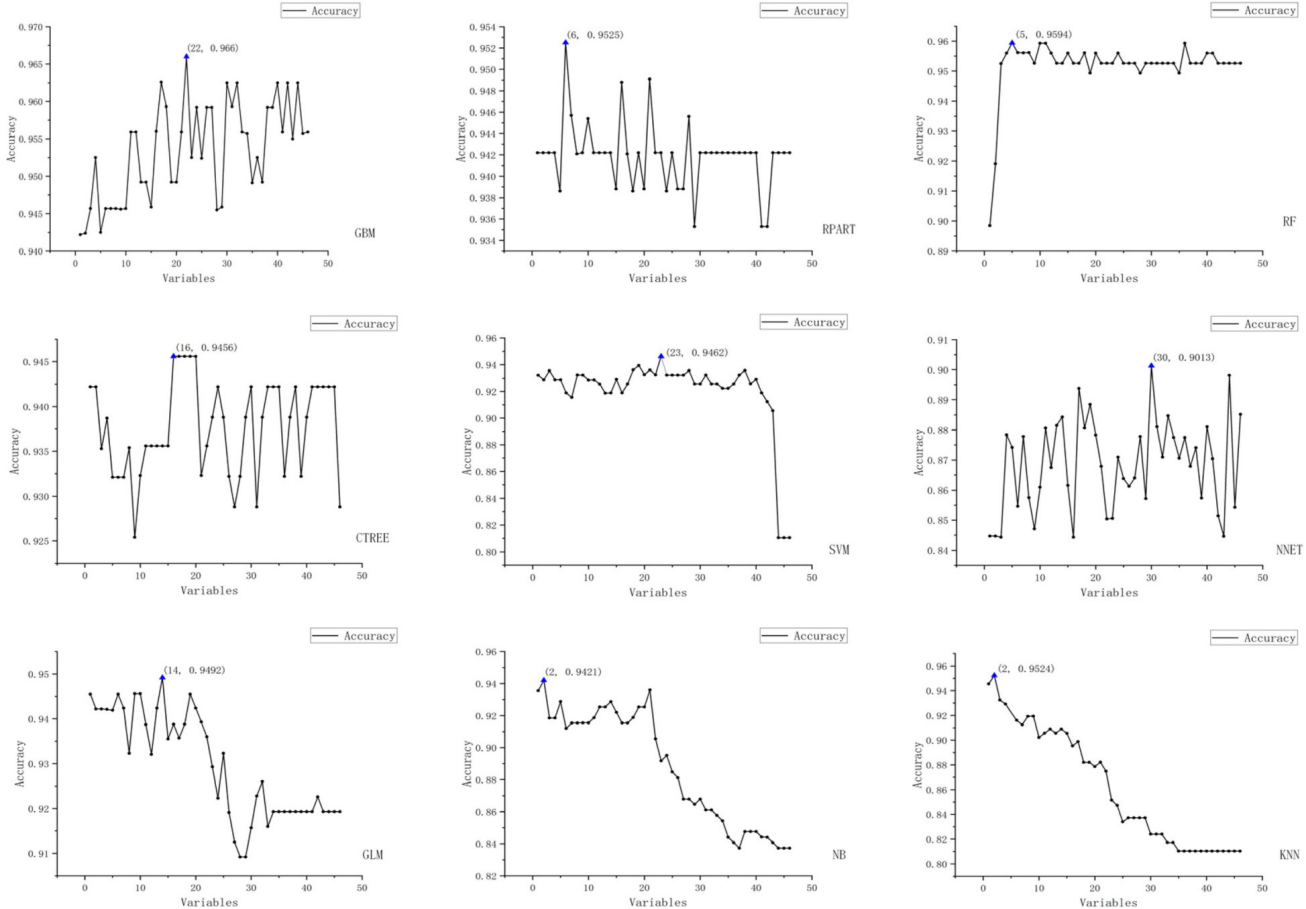
Schematic representation of recursive feature elimination (RFE) for nine machine learning models (Acc).

**Figure 4:**
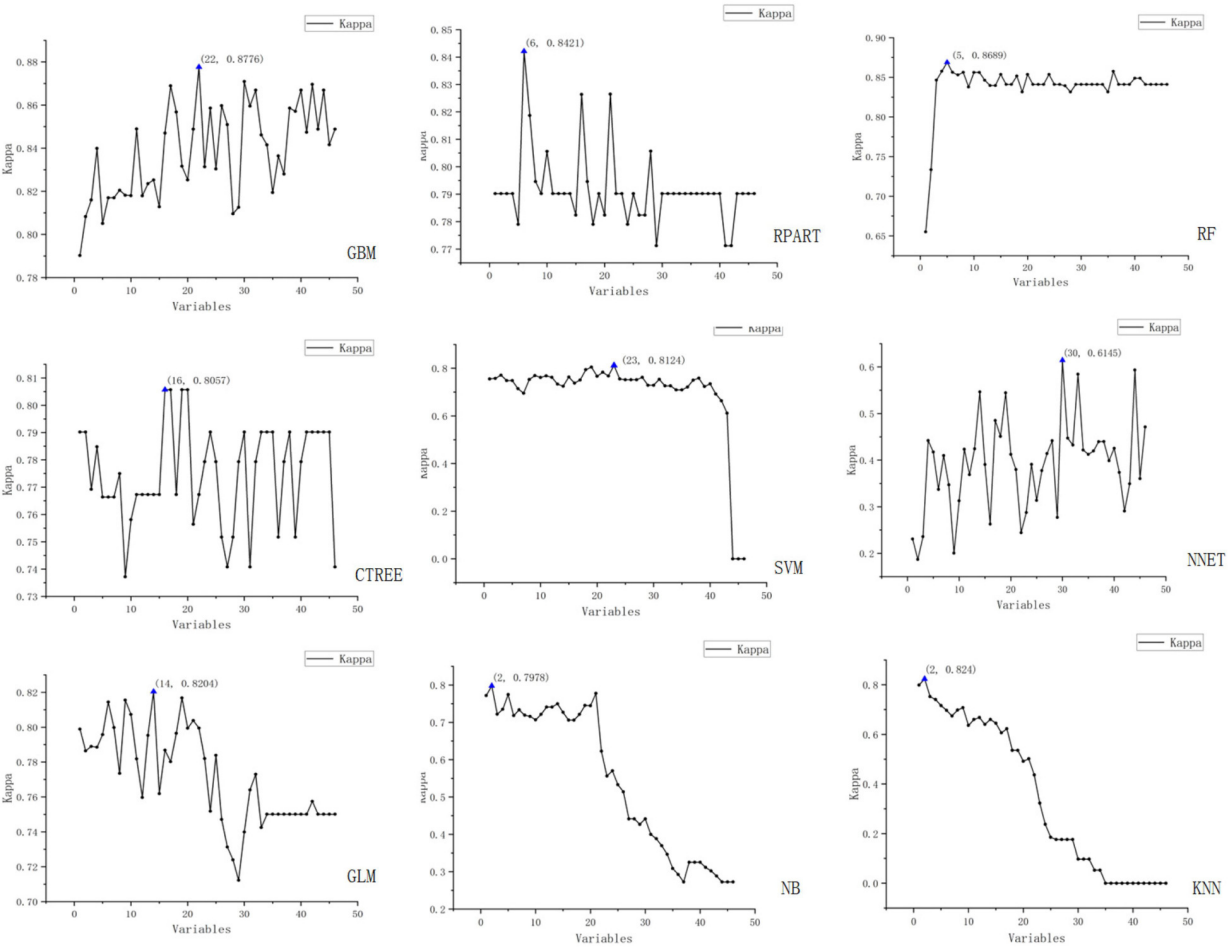
Schematic representation of recursive feature elimination (RFE) for nine machine learning models (Kappa).

We employed the Recursive Feature Elimination (RFE) strategy for feature selection, determining the optimal feature subset for each machine learning prediction model. The RFE variable selection process for each model is visualized in Figures 3, 4.

The figure displays the results of recursive feature elimination for nine different machine learning models. Each plot shows the relationship between the number of variables and cross-validated accuracy/Kappa values, where the *x*-axis represents the number of variables and the *y*-axis denotes the accuracy and Kappa values obtained via cross-validation.

#### 3.2.3 Model development and performance comparison

The machine learning models constructed after feature screening showed significant differences in performance between the training and test sets (Table 3).

**Table 3:** Comparison of the performance parameters of nine machine learning models.

Models	ACC	AUC	Sen	Spe	PPV	NPV	F1
Training set							
SVM	0.98	0.99	0.99	0.95	0.99	0.96	0.99
RF	0.99	0.99	1.00	0.98	0.99	1.00	0.999
KNN	0.96	0.98	0.99	0.82	0.96	0.96	0.98
CTREE	0.96	0.96	0.97	0.91	0.98	0.86	0.97
NB	0.95	0.96	0.97	0.82	0.96	0.88	0.97
RPART	0.96	0.95	0.96	0.93	0.98	0.85	0.97
GLM	0.97	0.99	0.99	0.88	0.97	0.94	0.98
NNET	0.96	0.94	0.97	0.89	0.97	0.88	0.97
GBM	1.00	1.00	1.00	1.00	1.00	1.00	1.00
Test set							
SVM	0.90	0.97	0.93	0.79	0.95	0.73	0.94
RF	0.92	0.97	0.97	0.71	0.93	0.83	0.95
KNN	0.89	0.91	0.97	0.57	0.90	0.80	0.93
CTREE	0.89	0.87	0.97	0.57	0.90	0.80	0.93
NB	0.89	0.94	0.97	0.57	0.90	0.80	0.93
RPART	0.89	0.80	0.97	0.57	0.90	0.75	0.93
GLM	0.86	0.90	0.92	0.64	0.92	0.64	0.92
NNET	0.86	0.80	0.93	0.57	0.90	0.67	0.92
GBM	0.92	0.94	0.95	0.79	0.95	0.79	0.95

**Figure 5:**
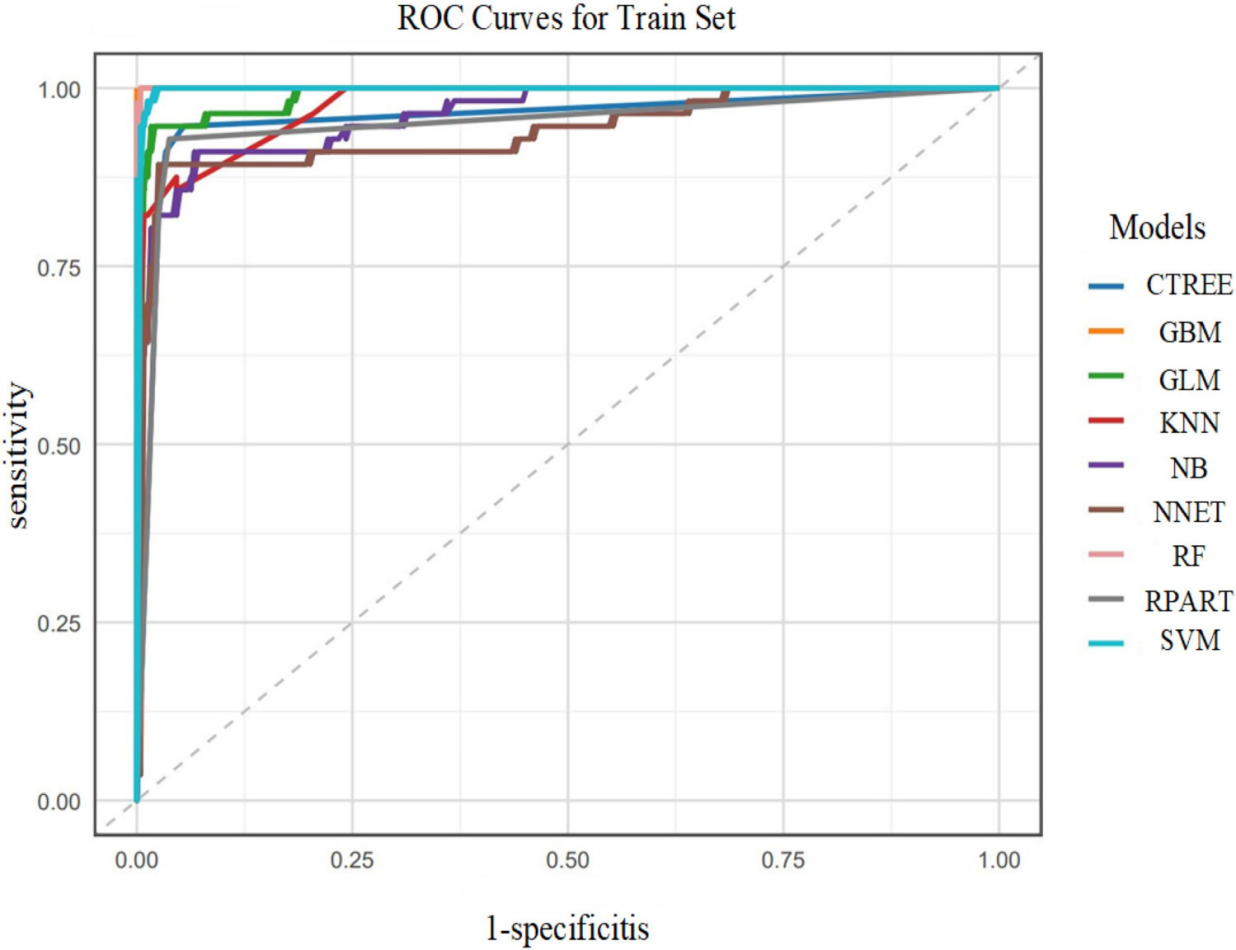
ROC curves of nine machine learning models in the training set.

In the training set, the GBM model showed optimal discrimination and calibration with an AUC of 1.0000. Concurrently, the RF model achieved the highest accuracy (1.0000), sensitivity (1.0000), specificity (1.0000), positive predictive value (PPV, 1.0000), negative predictive value (NPV, 1.0000), and F1 score (1.0000). The Random Forest (RF) model emerged as the top performer in the training set with 100% accuracy, sensitivity, specificity, and F1 score (1.0000), though its perfect performance suggests a potential risk of overfitting as shown in Figure 5.

**Figure 6:**
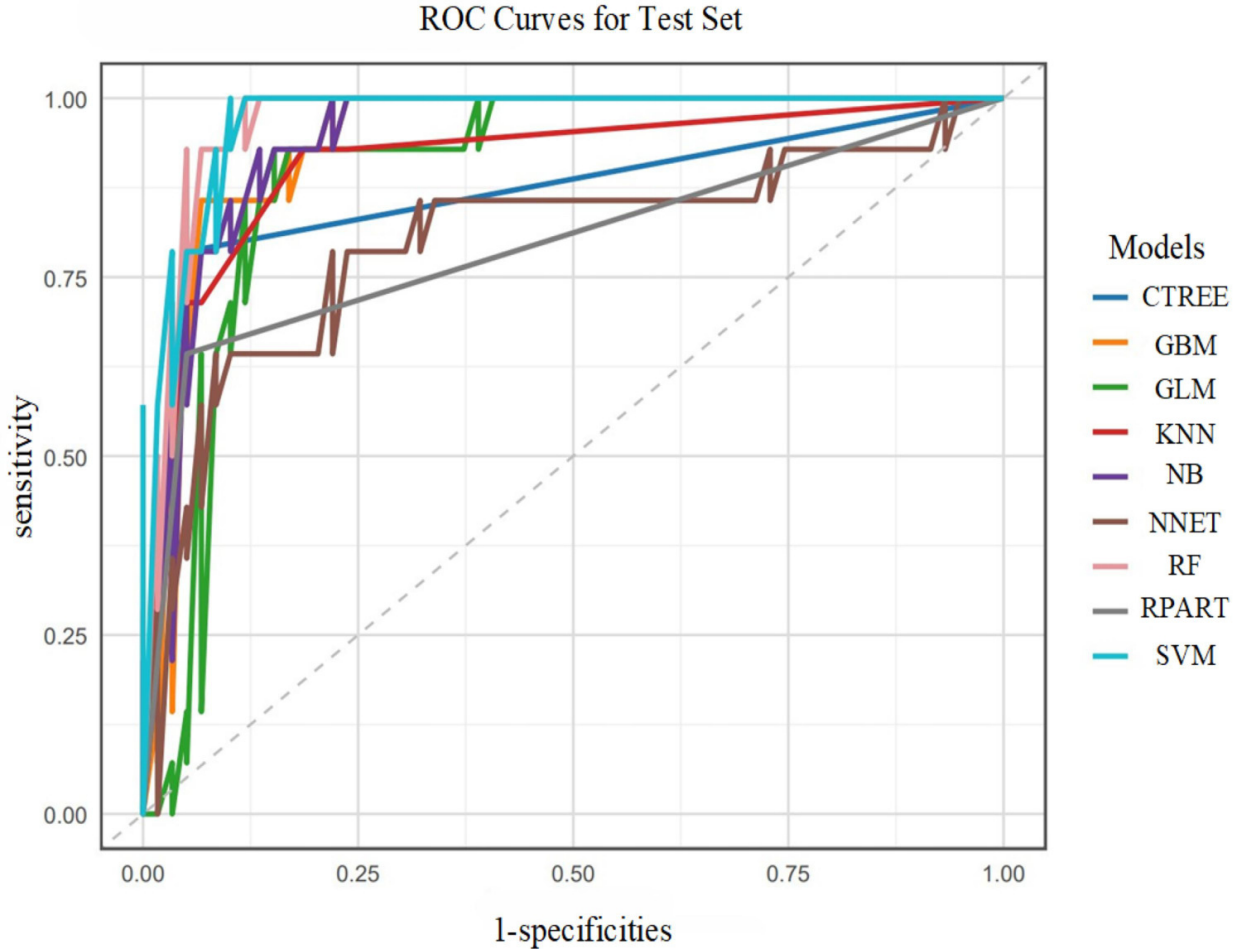
ROC curves of nine machine learning models in the test set.

In the test set, the SVM model exhibited the best performance, with an AUC of 0.9734. Correspondingly, the SVM model also showed relatively high accuracy (0.9041), sensitivity (0.9322), specificity (0.7857), PPV (0.9483), NPV (0.7333), and F1 score (0.9402). Additionally, the RF and GBM models ranked among the top in predictive performance: the RF model had an AUC of 0.9697, accuracy of 0.9178, sensitivity of 0.9661, specificity of 0.7143, PPV of 0.9344, NPV of 0.8333, and F1 score of 0.9500; the GBM model had an AUC of 0.9431, accuracy of 0.9178, sensitivity of 0.9492, specificity of 0.7857, PPV of 0.9492, NPV of 0.7857, and F1 score of 0.9492 as shown in Figure 6.

In summary, the SVM, RF, and GBM models were identified as the top three performing models for predicting the risk of postoperative AF in lung cancer patients.

#### 3.2.4 Development of the srg-LCPOAF model

**Figure 7:**
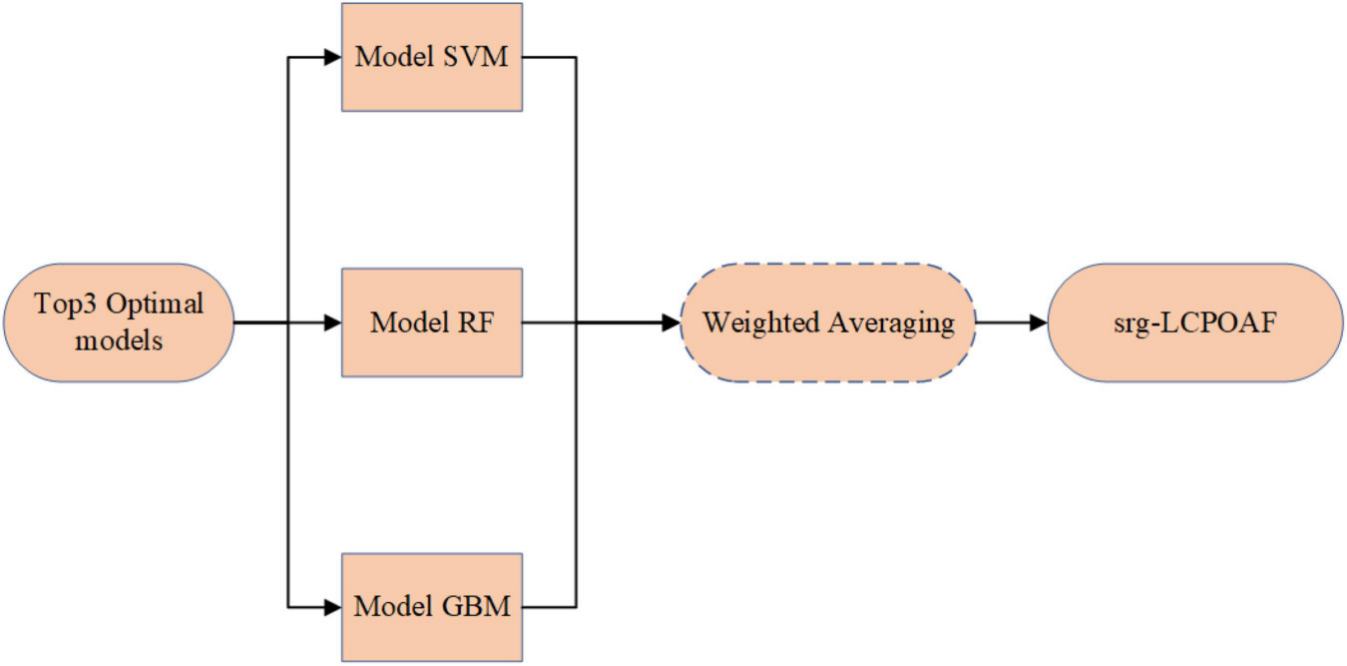
Integration model construction flow chart.

Based on the comparison of performance parameters (primarily ACC values) of the nine machine learning prediction models, the top three models with the best predictive performance were identified as the SVM, RF, and GBM models. An ensemble of these three models was then performed by assigning weights proportional to their ACC values on the test set (the workflow is shown in Figure 7), ultimately forming the integrated model srg-LCPOAF.

#### 3.2.5 Performance interpretation of the srg-LCPOAF model

Table 4 compares the performance of single models (SVM, RF, GBM) and the ensemble model srg-LCPOAF across seven evaluation metrics.

**Table 4:** Comparison of performance parameters between Top3's model and the ensemble model.

Models	ACC	AUC	Sen	Spe	PPV	NPV	F1
SVM	0.90	0.97	0.93	0.79	0.95	0.73	0.94
RF	0.92	0.97	0.97	0.71	0.93	0.83	0.95
GBM	0.92	0.94	0.95	0.79	0.95	0.79	0.95
srg-LCPOAF	0.95	0.97	0.97	0.86	0.97	0.86	0.97

Results showed that the ensemble model srg-LCPOAF outperformed all single models across all metrics, with significant improvements in ACC, specificity (Spe), NPV, and F1 score. RF and GBM exhibited similar performance, though RF had higher sensitivity (Sen = 0.97) and GBM showed better specificity (Spe = 0.79). While SVM demonstrated excellent AUC (0.97), its lower ACC and NPV indicated weaker discriminative ability for negative samples.

**Figure 8:**
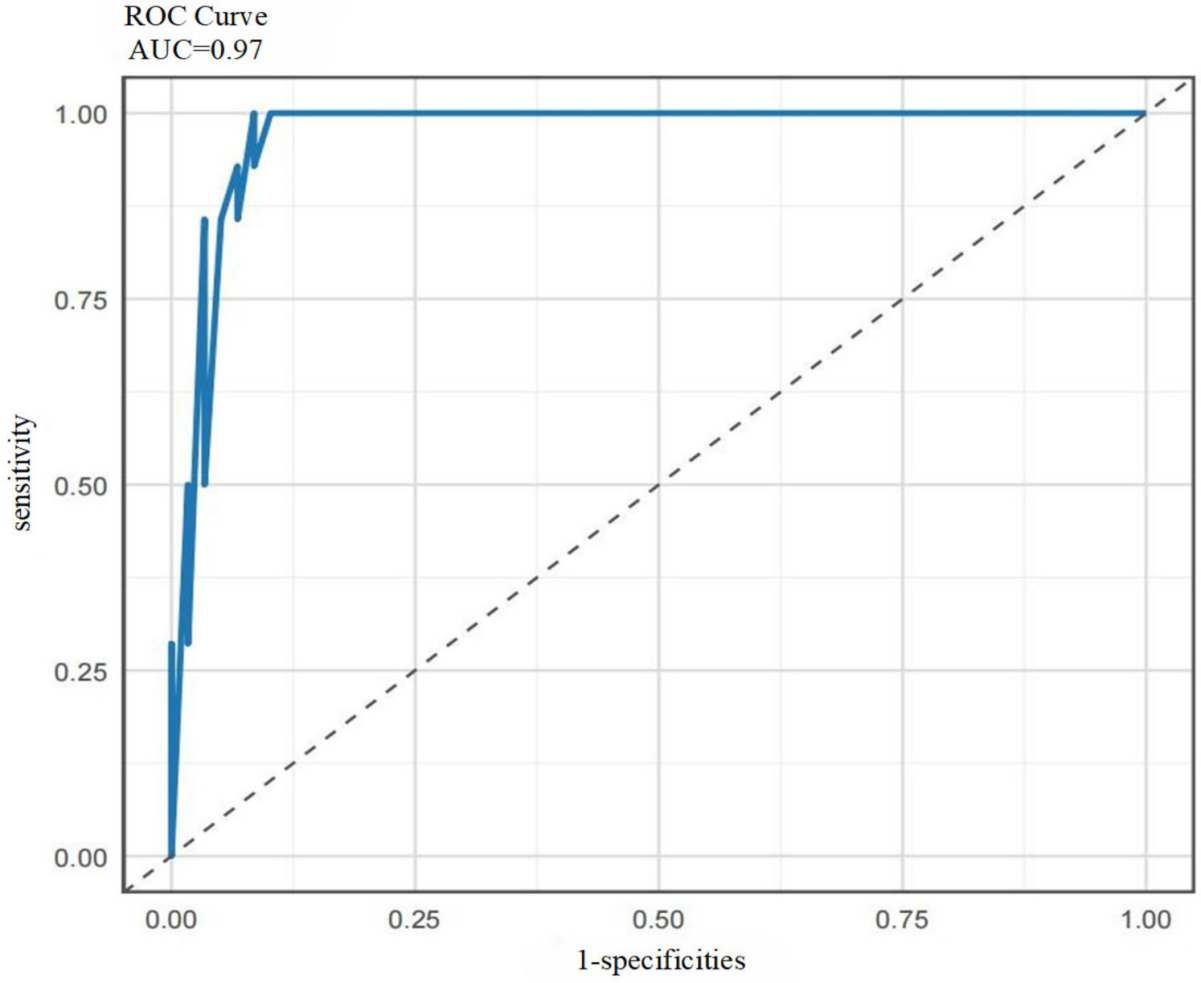
ROC curve of the ensemble model srg-LCPOAF.

Based on the ROC curve analysis of the ensemble model srg-LCPOAF (Figure 8), the curve rapidly ascends from the lower-left corner (0,0) to the upper-left corner (0,1) and then extends rightward to (1,1), indicating that the model achieves high sensitivity (Sen) at low false positive rates (1-Spe) and exhibits excellent classification performance. With an AUC value of 0.97, the model demonstrates stable discrimination between positive and negative samples across different thresholds and strong robustness to noise or variations in data distribution.

In summary, the predictive performance of the ensemble model srg-LCPOAF significantly outperforms any single model, highlighting its strong clinical predictive value.

## 4 Discussion

Postoperative atrial fibrillation (POAF) is a common and severe complication after lung cancer surgery, which is closely associated with surgical trauma, inflammatory response, autonomic nervous dysfunction, and patients' baseline cardiovascular status (7). POAF not only prolongs hospital stay and increases medical costs but also significantly correlates with adverse events such as stroke and heart failure (29). Therefore, constructing a high-precision risk prediction model is of great clinical value for early identification of high-risk patients and optimization of perioperative management strategies.

This study constructed nine machine learning models based on multi-dimensional clinical data to analyze and predict the risk of postoperative AF in lung cancer patients. During this process, we identified independent risk factors associated with POAF, fully interpreted and compared the performance of the nine predictive models, screened out the top three models with the best predictive performance, and finally developed an ensemble model for predicting POAF risk using ensemble learning. Results demonstrated that the ensemble model's enhanced predictive performance facilitated high-risk patient screening, enabling timely interventions to reduce hospitalization duration and improve quality of life.

This study found that the SVM, RF, and GBM models showed the best performance in predicting POAF risk (ACC values of 0.90, 0.92, and 0.92, respectively), significantly outperforming other models such as KNN (ACC = 0.89) and GLM (ACC = 0.86). This result may be related to the adaptability between the kernel function of SVM and the nonlinear pathological characteristics of POAF (30). SVM uses kernel function to implicitly map the non-linearly separable problem in the original low-dimensional space to a high-dimensional feature space, so that the data becomes linearly separable in the space. However, the occurrence of POAF also involves multi-system interactions: surgical trauma activates systemic inflammatory responses (such as increased IL-6 and CRP), inflammatory factors damage atrial myocytes through oxidative stress (11); intraoperative thoracic operations may directly stretch the pericardium, inducing autonomic nervous imbalance (31); postoperative electrolyte disorders (hypokalemia, hypomagnesemia) further aggravate electrophysiological instability [reference]. The interactions among these factors (such as the synergistic effect of CRP and serum potassium concentration) are highly nonlinear, and the radial basis function (RBF) kernel of SVM can effectively capture such complex associations through its local sensitivity. Studies have also shown that this result may be related to the strong compatibility between the ensemble decision-making of RF and the multi-factor heterogeneity of POAF. The random feature selection of RF reduces the interference of redundant variables (such as collinearity between systolic blood pressure and mean arterial pressure) and identifies key driving factors through Gini importance scores (32).

It is noteworthy that the occurrence of atrial fibrillation is not only associated with perioperative factors such as preoperative test results, cardiopulmonary function indicators, and surgical procedures, but also frequently arises secondary to various underlying diseases and long-term lifestyle factors. In addition to clinical conditions such as hypertension, structural heart disease, and hyperthyroidism, long-term high-intensity endurance exercise has also been identified as an independent risk factor for atrial fibrillation and atrial flutter. Among various cardiovascular risk factors, hypertension is one of the most common comorbidities associated with atrial fibrillation, present in approximately 40% of patients with atrial fibrillation. Furthermore, attribution risk studies indicate that a history of hypertension may account for approximately 24% of atrial fibrillation cases (33). Therefore, for patients with atrial fibrillation and concomitant hypertension, emphasis should be placed on standardized blood pressure management. Appropriate antihypertensive medications should be selected based on the patient's specific circumstances to improve long-term prognosis. Hyperthyroidism not only increases the risk of worsening underlying heart disease but also directly induces structural and functional changes in the heart. Atrial fibrillation is a common cardiac manifestation in hyperthyroidism. Reports indicate that the prevalence of atrial fibrillation in hyperthyroidism patients ranges from 1% to 60%, depending on gender, age, and the presence of prior or concomitant cardiovascular disease (34). Although the protective effects of regular, appropriate exercise on the cardiovascular system are widely recognized, the long-term impact of prolonged, high-intensity endurance activities—such as marathons and long-distance cycling—on the heart remains to be thoroughly investigated. Research indicates that such exercises may be associated with the development of atrial fibrillation and atrial flutter through mechanisms such as promoting atrial enlargement and increasing the risk of myocardial fibrosis (35). Although most lung cancer patients are not professional athletes, their preoperative physical fitness and cardiopulmonary reserve capacity may have a potential association with exercise endurance, warranting attention in risk assessment. Additionally, unhealthy lifestyle habits such as heavy alcohol consumption and smoking increase susceptibility to atrial fibrillation by promoting atrial electrical and structural remodeling, thereby contributing to its onset (36). If patients exhibit autonomic dysregulation or have underlying cardiomyopathy, these should not be overlooked as potential triggers for arrhythmia. In structural heart disease, hypertrophic cardiomyopathy (HCM) is a major cause of atrial fibrillation (AF). Patients with HCM exhibit a significantly higher prevalence of AF compared to the general population, and this condition is closely associated with pathological changes such as left atrial remodeling, fibrosis, and left ventricular outflow tract obstruction. Even with low CHA₂DS₂-VASc scores, these patients exhibit markedly elevated thromboembolic risk, underscoring the independent impact of underlying cardiac structural abnormalities on AF occurrence and stroke risk. Therefore, when assessing AF risk—particularly in patients with structural heart disease—it is essential to incorporate disease-specific factors into the overall risk evaluation framework. This includes considering atrial myocardial disease associated with cardiomyopathies such as HCM (37). Research indicates that autonomic dysregulation, such as excessive vagal activation, may increase susceptibility to atrial fibrillation by altering atrial electrophysiological properties. This mechanism has been further corroborated in studies investigating reflex syncope in patients with Brugada syndrome (38). Among lung cancer patients, due to the prevalence of long-term smoking histories and the frequent coexistence of underlying cardiopulmonary conditions, the atrial myocardium is more susceptible to electrical activity disturbances following acute stressors such as surgical trauma, thereby increasing the risk of postoperatively acquired atrial fibrillation (POAF). Therefore, in the future optimization of predictive models, integrating such underlying diseases, lifestyle indicators, and functional assessment parameters will facilitate the development of a more comprehensive and precise multidimensional risk assessment system.

So far, multiple studies have been conducted on risk factor analysis and prediction model construction for POAF in lung cancer patients. Early domestic studies mostly used traditional methods such as logistic regression and Cox proportional hazards model. For example, in recent years, some tertiary hospitals have begun to attempt machine learning models. For instance, a 2022 study from Shanghai Chest Hospital used random forest (RF) to integrate preoperative cardiac ultrasound (left ventricular ejection fraction, LAD) and postoperative inflammatory markers (CRP, IL-6), increasing the AUC to 0.82 (39), but the sample size was small (*n* = 300) and lacked external validation. Related studies in Europe and America introduced machine learning earlier. For example, a 2021 study from Mayo Clinic used a gradient boosting machine (GBM) to integrate preoperative NT-proBNP, intraoperative fluid balance, and early postoperative electrocardiogram monitoring data, achieving an AUC of 0.87, which was validated in the European Society of Thoracic Surgery (ESTS) database (40). A 2023 study on the NCDB database of the National Cancer Institute (NCI) of the United States combined electronic health records (EHR), Holter monitoring, and genomic data (such as miRNA-21 expression) to construct an XGBoost model, with an AUC exceeding 0.90, but the computational complexity was high, making clinical implementation difficult (41). However, existing studies have significant limitations. First, most studies rely on single-center retrospective data (sample size <1,000), leading to selection bias and information missing in the research; second, the external validation rate in the studies is low, which significantly reduces the accuracy and practical guiding significance of the research results; furthermore, most existing studies stop at the interpretation of the best predictive model, but do not continue to study the further application of the model.

However, in this study, after comparing the nine predictive models, we screened out the top three models with the best predictive performance, namely SVM, RF, and GBM. The predictive performance of these three models was integrated using an ensemble strategy to form the ensemble model srg-LCPOAF. The ACC of this ensemble model reached 0.95, with sensitivity and specificity of 0.97 and 0.86, respectively, representing a 4% and 7% improvement over the best single model. This finding indicates that the establishment of the ensemble model has improved various performance metrics of the model to a certain extent, enabling clinicians to more accurately predict whether lung cancer patients will develop postoperative AF, precisely identify high-risk patients, and take preventive measures in advance.

Similarly, this study also has many shortcomings. First, the data source is single: single-center retrospective data may lead to selection bias in the data; second, emerging biomarkers are not included: such as microRNA (miR-21, miR-208a) and gene polymorphisms (PITX2 rs2200733). Including the above characteristic variables may improve the prediction accuracy; finally, the dynamic prediction ability is insufficient: the existing characteristic variables mainly include preoperative or static postoperative data, lacking the integration of real-time intraoperative indicators (such as SVV, BIS monitoring) and continuous postoperative time-series data. In the future, based on the current study, we can add multi-center prospective studies, which can avoid errors caused by information bias and selection bias to a certain extent. At the same time, we can also combine instrumental variables (such as Mendelian randomization) to distinguish confounding factors (such as smoking history) from causal drivers, enhancing the causal inference theory of postoperative AF.

In conclusion, this study developed nine machine learning-based risk prediction models for postoperative atrial fibrillation (POAF) in lung cancer patients. A stacking ensemble integrating SVM, random forest (RF), and GBM algorithms demonstrated superior predictive performance, enhancing both accuracy and clinical utility for POAF risk assessment after pulmonary resection. This model not only provides a reliable tool for individualized risk stratification but also sets a methodological paradigm for multi-model fusion strategies in complex medical scenarios. In the future, it is necessary to further promote the transformation from the prediction model to a clinical decision support system, ultimately achieving patient-centered precision perioperative management.

## Data Availability

The raw data supporting the conclusions of this article will be made available by the authors, without undue reservation.

## References

[1] Bonaca MP Olenchock BA Salem JE Wiviott SD Ederhy S Cohen A Myocarditis in the setting of cancer therapeutics: proposed case definitions for emerging clinical syndromes in cardio-oncology. Circulation. (2019) 140(2):80–91. 10.1161/circulationaha.118.034497 31390169 PMC6779326

[2] Chinese Thoracic Society, Chinese Medical Association; Chinese Alliance Against Lung Cancer Expert Group. Chinese expert consensus on diagnosis and treatment of pulmonary nodules (2024). Zhonghua Jie He He Hu Xi Za Zhi. (2024) 47(8):716–29. 10.3760/cma.j.cn112147-20231109-00300 39069848

[3] Han B Zheng R Zeng H Wang S Sun K Chen R Cancer incidence and mortality in China, 2022. J Natl Cancer Cent. (2024) 4(1):47–53. 10.1016/j.jncc.2024.01.006 39036382 PMC11256708

[4] Wu F Wang L Zhou C. Lung cancer in china: Current and prospect. Curr Opin Oncol. (2021) 33(1):40–6. 10.1097/CCO.0000000000000703 33165004

[5] Zhao D Lu J Zeng W Zhang C You Y. Changing trends in disease burden of lung cancer in china from 1990 to 2019 and following 15-year prediction. Curr Probl Cancer. (2024) 48:101036. 10.1016/j.currproblcancer.2023.101036 37926577

[6] Zhou Y Wen Y Xiang Z Ma J Lin Y Huang Y Cancer survival trends in southeastern china, 2011–2021: a population-based study. Clin Epidemiol. (2024) 16:45–56. 10.2147/CLEP.S442152 38318284 PMC10840559

[7] Gaudino M Di Franco A Rong LQ Piccini J Mack M. Postoperative atrial fibrillation: from mechanisms to treatment. Eur Heart J. (2023) 44(12):1020–39. 10.1093/eurheartj/ehad019 36721960 PMC10226752

[8] Bandyopadhyay D Ball S Hajra A Chakraborty S Dey AK Ghosh RK Impact of atrial fibrillation in patients with lung cancer: insights from national inpatient sample. Int J Cardiol Heart Vasc. (2019) 22:216–7. 10.1016/j.ijcha.2019.02.012 30963100 PMC6437280

[9] Cao M Chen W. Epidemiology of lung cancer in china. Thorac Cancer. (2019) 10(1):3–7. 10.1111/1759-7714.12916 30485694 PMC6312841

[10] Hashimoto H Usui G Tsugeno Y Sugita K Amori G Morikawa T Cerebral thromboembolism after lobectomy for lung cancer: pathological diagnosis and mechanism of thrombus formation. Cancers. (2019) 11(4):488. 10.3390/cancers11040488 30959839 PMC6521235

[11] Semeraro GC Meroni CA Cipolla CM Cardinale DM. Atrial fibrillation after lung cancer surgery: prediction, prevention and anticoagulation management. Cancers. (2021) 13(16):4012. 10.3390/cancers13164012 34439166 PMC8394120

[12] Teh R Kerse N Pillai A Lumley T Rolleston A Kyaw TA Atrial fibrillation incidence and outcomes in two cohorts of octogenarians: lilacs nz. BMC Geriatr. (2023) 23(1):197. 10.1186/s12877-023-03902-5 36997900 PMC10064671

[13] Allan KS Henry S Aves T Banfield L Victor JC Dorian P Comparison of health-related quality of life in patients with atrial fibrillation treated with catheter ablation or antiarrhythmic drug therapy: a systematic review and meta-analysis protocol. BMJ Open. (2017) 7(8):e017577. 10.1136/bmjopen-2017-017577 PMC5724133 28827273

[14] San TMM Han KPP Ismail M Thu LM Thet MS. Pericardiotomy and atrial fibrillation after isolated coronary artery bypass grafting: a systematic review and meta-analysis of 16 randomised controlled trials. Cardiovasc Revasc Med. (2024) 66:27–32. 10.1016/j.carrev.2024.03.023 38584081

[15] Muranishi Y Sonobe M Menju T Aoyama A Chen-Yoshikawa TF Sato T Atrial fibrillation after lung cancer surgery: incidence, severity, and risk factors. Surg Today. (2017) 47(2):252–8. 10.1007/s00595-016-1380-y 27382978

[16] Jiang J He M Xu Y. Preoperative electrocardiogram and perioperative methods for predicting new-onset atrial fibrillation during lung surgery. J Cardiothorac Vasc Anesth. (2021) 35(5):1424–30. 10.1053/j.jvca.2020.09.107 33041171

[17] Zhang L Li X Wu H Luo J. Risk factors associated with atrial fibrillation following lung cancer surgery: a multi-center case-control study. Asian J Surg. (2024) 47(1):176–83. 10.1016/j.asjsur.2023.06.108 37419802

[18] Zuo K Li J Li K Hu C Gao Y Chen M Disordered gut microbiota and alterations in metabolic patterns are associated with atrial fibrillation. Gigascience. (2019) 8(6):giz058. 10.1093/gigascience/giz058 31149718 PMC6543127

[19] Tong C Niu Z Zhu H Li T Xu Y Yan Y Development and external validation of a novel model for predicting new clinically important atrial fibrillation after thoracoscopic anatomical lung cancer surgery: a multicenter retrospective cohort study. Int J Surg. (2024) 110(3):1645–52. 10.1097/JS9.0000000000001006 38181118 PMC10942185

[20] Tohidinezhad F Nürnberg L Vaassen F Ma Ter Bekke R Jwl Aerts H El Hendriks L Prediction of new-onset atrial fibrillation in patients with non-small cell lung cancer treated with curative-intent conventional radiotherapy. Radiother Oncol. (2024) 201:110544. 10.1016/j.radonc.2024.110544 39341504

[21] Van Gelder IC Rienstra M Bunting KV Casado-Arroyo R Caso V Crijns HJGM 2024 ESC guidelines for the management of atrial fibrillation developed in collaboration with the european association for cardio-thoracic surgery (EACTS). Eur Heart J. (2024) 45(36):3314–414. 10.1093/eurheartj/ehae176 39210723

[22] Ortiz BL Gupta V Kumar R Jalin A Cao X Ziegenbein C Data preprocessing techniques for ai and machine learning readiness: scoping review of wearable sensor data in cancer care. JMIR Mhealth Uhealth. (2024) 12:e59587. 10.2196/59587 38626290 PMC11470224

[23] Heymans MW Twisk JWR. Handling missing data in clinical research. J Clin Epidemiol. (2022) 151:185–8. 10.1016/j.jclinepi.2022.08.016 36150546

[24] Blázquez-García A Conde A Mori U Lozano JA. A review on outlier/anomaly detection in time series data. ACM Comput Surv. (2022) 54(3):1–36. 10.1145/3444690

[25] Staartjes VE Kernbach JM Stumpo V van Niftrik CHB Serra C Regli L. Foundations of Feature Selection in Clinical Prediction Modeling. Cham: Springer International Publishing (2022). p. 51–7. 10.1007/978-3-030-85292-4_7 34862527

[26] Lausser L Szekely R Schmid F Maucher M Kestler H. Efficient cross-validation traversals in feature subset selection. Sci Rep. (2022) 12(1):21485. 10.1038/s41598-022-25942-4 36509882 PMC9744898

[27] Kumar V Prabha C Gupta D Juneja S Kumari S Nauman A. Multi-model machine learning framework for lung cancer risk prediction: a comparative analysis of nine classifiers with hybrid and ensemble approaches using behavioral and hematological parameters. SLAS Technology. (2025) 33:100314. 10.1016/j.slast.2025.100314 40562280

[28] Rainio O Teuho J Klén R. Evaluation metrics and statistical tests for machine learning. Sci Rep. (2024) 14(1):6086. 10.1038/s41598-024-56706-x 38480847 PMC10937649

[29] Meenashi Sundaram D Vasavada AM Ravindra C Rengan V Meenashi Sundaram P. The management of postoperative atrial fibrillation (poaf): a systematic review. Cureus. (2023) 15(8):e42880. 10.7759/cureus.42880 37664333 PMC10474445

[30] Amar D Piening D Zhang H Tan KS Desiderio D. P15—clinical prediction model for postoperative atrial fibrillation (poaf). J Cardiothorac Vasc Anesth. (2016) 30:S19. 10.1053/j.jvca.2016.03.060

[31] Yang Z Tiemuerniyazi X Huang S Song Y Xu F Feng W. Partial cardiac denervation to prevent postoperative atrial fibrillation after coronary artery bypass grafting: the pcad-poaf randomized clinical trial. Am J Cardiol. (2024) 221:120–5. 10.1016/j.amjcard.2024.04.018 38649126

[32] Liu Y Du Z Lu Y Ma Y Yang Y Osmanaj F Gut microbiota metabolism disturbance is associated with postoperative atrial fibrillation after coronary artery bypass grafting. npj Cardiovascular Health. (2024) 1(1):5. 10.1038/s44325-024-00003-z PMC12912376 41775960

[33] Middeldorp ME Ariyaratnam JP Kamsani SH Albert Christine M Sanders P. Hypertension and atrial fibrillation. J Hypertens. (2022) 40(12):2337–52. 10.1097/hjh.0000000000003278 36204994

[34] Frost L Vestergaard P Mosekilde L. Hyperthyroidism and risk of atrial fibrillation or flutter: a population-based study. Arch Intern Med. (2004) 164(15):1675–8. 10.1001/archinte.164.15.1675/15302638

[35] Mont L Elosua R Brugada J. Endurance sport practice as a risk factor for atrial fibrillation and atrial flutter. Europace. (2008) 11(1):11–7. 10.1093/europace/eun289 18988654 PMC2638655

[36] Chamberlain AM Agarwal SK Folsom AR Duval S Soliman EZ Ambrose M Smoking and incidence of atrial fibrillation: results from the atherosclerosis risk in communities (ARIC) study. Heart Rhythm. (2011) 8(8):1160–6. 10.1016/j.hrthm.2011.03.038 21419237 PMC3139831

[37] Choi YJ Lakdawala NK. Atrial fibrillation and thromboembolic risk in hypertrophic cardiomyopathy. J Cardiovasc Imaging. (2025) 33:12. 10.1186/s44348-025-00057-2 40855573 PMC12376757

[38] Mascia G Barca L Brugada J Arbelo E Monaco C Vlachos K Characterization of reflex syncope in brugada syndrome: a literature review. J Cardiovasc Med. (2025) 26(8):454–61. 10.2459/JCM.0000000000001758 40747706

[39] Qian L Zhou Y Zeng W Chen X Ding Z Shen Y A random forest algorithm predicting model combining intraoperative frozen section analysis and clinical features guides surgical strategy for peripheral solitary pulmonary nodules. Transl Lung Cancer Res. (2022) 11(6):1132–44. 10.21037/tlcr-22-395 35832446 PMC9271446

[40] Pezel T Toupin S Bousson V Hamzi K Hovasse T Lefevre T A machine learning model using cardiac CT and MRI data predicts cardiovascular events in obstructive coronary artery disease. Radiology. (2025) 314(1):e233030. 10.1148/radiol.233030 39807980

[41] Maturi B Dulal S Sayana SB Ibrahim A Ramakrishna M Chinta V Revolutionizing cardiology: the role of artificial intelligence in echocardiography. J Clin Med. (2025) 14:625. 10.3390/jcm14020625 39860630 PMC11766369

