# Correction: Construction of a risk prediction model for postoperative atrial ﬁbrillation in lung cancer patients based on multidimensional feature fusion and ensemble learning

**DOI:** 10.3389/fcvm.2025.1743452

**Published:** 2025-11-26

**Authors:** Ziwei Gong, Silamuguli Haierla, Jing Shi, Xinya Liu

**Affiliations:** 1 College of Public Health, Xinjiang Medical University, Urumqi, China; 2 Department of Oncology and Cardiology, Affiliated Cancer Hospital of Xinjiang Medical University, Urumqi, China; 3 The Fifth Affiliated Hospital of Xinjiang Medical University, Urumqi, China

**Keywords:** lung cancer, machine learning, postoperative atrial fibrillation, risk factors, prediction model

Author Silamuguli Haierla was erroneously spelled as Slamuguli Hella.

The original version of this article has been updated.

